# The silent pandemic in South Africa: Extra-pulmonary tuberculosis from head to heel

**DOI:** 10.4102/sajr.v25i1.2026

**Published:** 2021-04-16

**Authors:** Camilla E. Le Roux, Sucari S.C. Vlok

**Affiliations:** 1 Department of Medical Imaging and Clinical Oncology, Medicine and Health Sciences, Tygerberg Hospital, Stellenbosch University, Cape Town, South Africa

**Keywords:** extra-pulmonary TB, circulatory system, lungs, organ systems, chest radiograph, diagnosis

## Abstract

Extra-pulmonary tuberculosis (EPTB), caused by *Mycobacterium tuberculosis*, is the leading cause of communicable disease-related deaths in people with human immunodeficiency virus (HIV) worldwide and in South Africa. *Mycobacterium tuberculosis* disseminates haematogenously from an active primary lung focus and may affect extra-pulmonary sites in up to 15% of patients. Extra-pulmonary TB may present with a normal chest radiograph, which often causes a significant diagnostic dilemma. This review describes the main sites of involvement in EPTB, which is illustrated by local imaging examples.

## Introduction

Worldwide and in South Africa, tuberculosis (TB), a communicable airborne disease caused by *Mycobacterium tuberculosis*, remains the leading cause of death, followed closely by circulatory system diseases. In addition, it is one of the main causes of mortality amongst patients with human immunodeficiency virus (HIV) infection worldwide.^1,2^

Tuberculosis predominantly affects the lungs; however, in up to 15% of patients, extra-pulmonary sites may be involved. Extra-pulmonary tuberculosis (EPTB) may be the result of haematogenous dissemination from an active primary focus in the lung to other organ system(s) in the body, and this may present years after the initial pulmonary infection.^3^ A normal chest radiograph or negative laboratory tests do not exclude EPTB, a diagnosis that necessitates a high index of suspicion, especially when the patient also tests HIV positive.^3,4^

This review briefly outlines the main radiological findings of TB in extra-pulmonary sites, of which pleural and lymph node involvement was found to be the most prevalent in this research setting.

## Central nervous system tuberculosis

Tuberculosis may involve the parenchyma, meninges or spine.

### Parenchyma

Parenchymal tuberculosis can result from direct spread of infection via the cerebrospinal fluid (CSF) or haematogenous dissemination.^5,6^

**FIGURE 1:**
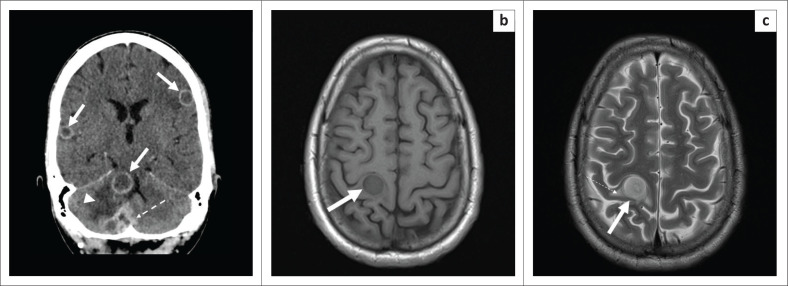
A 28-year-old female with disseminated tuberculosis who presented with a 3-month history of weakness and horizontal nystagmus. (a) Axial post-contrast computed tomography brain through the cerebellum reveals several hypodense supra- and infra-tentorial rim-enhancing tuberculomas (white solid arrows) of which some are clustered (white arrow with dashed line) with surrounding oedema (white arrow head). (b) Axial magnetic resonance imaging of the brain through the centrum semiovale of the same patient reveals a non-caseating granuloma, T1-weighted hypointense (white solid arrow) and (c) T2-weighted hyperintense (white solid arrow) with minimal surrounding oedema (white arrow with dashed line). Tuberculosis was confirmed on cerebrospinal fluid analysis.

A tuberculoma – also known as the TB granuloma – is the most frequent manifestation of parenchymal involvement, with both computed tomography (CT) and magnetic resonance imaging (MRI) signal characteristics depending on the stage of infection. Tuberculomas, whether caseating or non-caseating, are usually surrounded by moderate to marked oedema on all modalities. Tuberculomas may be hypo- or hyperdense on uncontrasted CT and reveal avid, homogeneous or rim enhancement on post-contrast CT (Figure 1a). At MRI, the non-caseating granulomas are T1-weighted (T1W) hypointense (Figure 1b) and T2-weighted (T2W) hyperintense (Figure 1c) with solid, homogeneous T1W post-gadolinium-contrast enhancement. A solid caseating granuloma is hypointense on both T1W and T2W sequences, and reveals ring enhancement. Caseating granulomas demonstrate diffusion restriction, with hyperintense signal at diffusion-weighted imaging (DWI) and corresponding hypointense signal on apparent diffusion coefficient map (ADC), because of their central viscous nature.^5,6,7^

A tuberculoma may rarely progress to a tuberculous abscess.^3^ A tuberculous abscess is usually solitary, can become very large, is often multiloculated with rim enhancement on post-contrast imaging and surrounded by marked oedema on all modalities. On MRI, a tuberculous abscess is hypointense on T1W, hyperintense with a hypointense rim on T2W and reveals rim enhancement on T1W post-gadolinium-contrasted imaging.^5,6,7^

Infarcts typically involve the deep grey nuclei, that is, basal ganglia, thalamus and internal capsule.^5,6,7^

### Meninges

**FIGURE 2:**
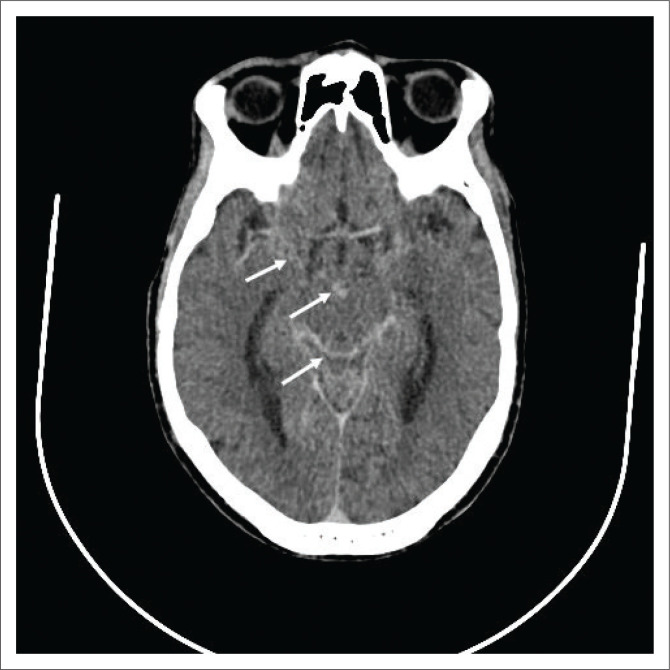
A 50-year-old patient with human immunodeficiency virus presented with new onset seizures and a reduced Glasgow coma scale (11/15). Post-contrasted computed tomography of the brain revealed diffuse leptomeningeal enhancement (white arrows) consistent with tuberculosis meningitis, which was confirmed based on cerebrospinal fluid analysis.

Meningeal spread occurs because of either rupture of a subpial focus or haematogenous spread via the meningeal vessels, resulting in a thick basal leptomeningeal exudate that enhances avidly on post-contrast imaging (Figure 2). Dural involvement with pachymeningitis is also possible.

The sequelae of meningeal involvement include hydrocephalus, infarcts, vasculitis and cranial nerve palsies. Hydrocephalus is caused by reduced CSF absorption at the level of the arachnoid villi. Cerebrospinal fluid analysis and typical imaging findings aid in distinguishing tuberculous meningitis from other infective aetiologies.^3,4,5^

### Spine

**FIGURE 3:**
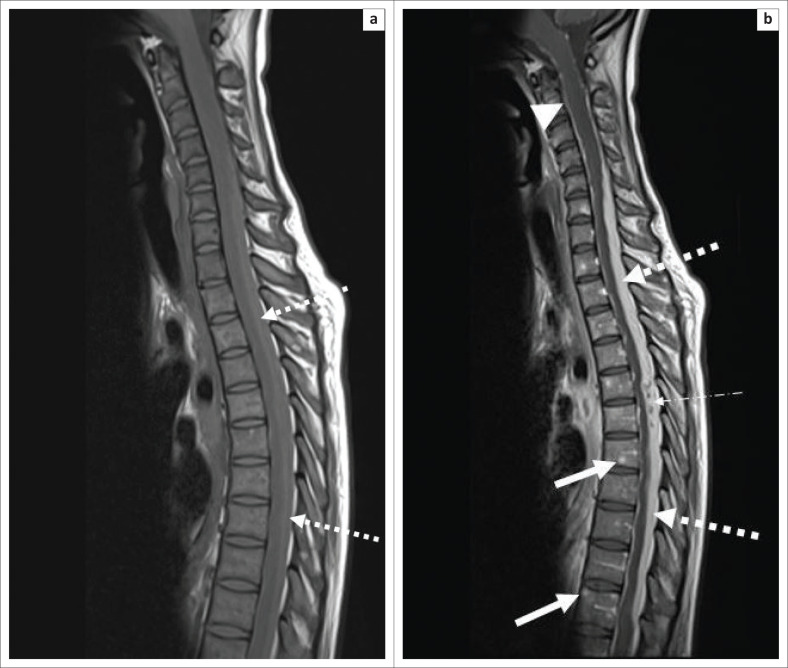
A 37-year-old female with advanced human immunodeficiency virus and disseminated tuberculosis presented with a 3-week history of bilateral lower limb weakness, urinary retention and loss of bowel control. (a) Sagittal T1-weighted pre-contrasted magnetic resonance imaging of the spine reveals inflammatory exudate along the posterior cord (white arrow with dashed line). (b) Sagittal T1-weighted post-contrasted magnetic resonance imaging of the spine reveals multiple enhancing microabscesses throughout the spine (solid white arrows), diffuse leptomeningeal enhancement along the brainstem and spinal cord (white arrow head) and long-segment enhancing inflammatory exudate along the posterior cord, which compresses the cord against the vertebral column (white arrows with dashed line) and contains central necrosis (white arrow with dotted dash line).

The bacilli spread haematogenously via Batson’s venous plexus or as a result of reactivation of dormant foci.^5^ In severe cases, multiple microabscesses may be scattered throughout the spine with involvement of the meninges (Figure 3).

The vertebral spine is the most common site of musculoskeletal involvement, with the lower thoracic and upper lumbar spine being the most frequently affected sites. Classic findings of tuberculous spondylitis (Pott disease) are contiguous involvement of more than one vertebral level, with a predilection for the anterior vertebral body adjacent to the end plates. Involvement of the posterior elements is rare in comparison with the end plate changes.^6,8^ Presentation with ivory vertebra or complete vertebral collapse (plana) is possible.

**FIGURE 4:**
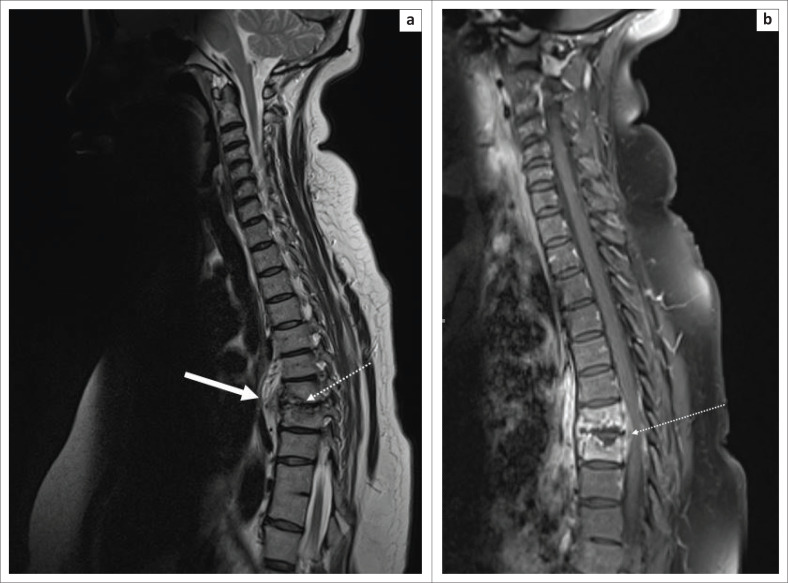
A 48-year-old human immunodeficiency virus-positive female who presented with progressive back pain. (a) Sagittal T2-weighted magnetic resonance imaging of the cervical and thoracic spine revealed spondylodiscitis centred at the T7/T8 level with destruction of the anteroinferior end plate of T7 and superoanterior end plate of T8 vertebrae with early destruction of the T7/T8 intervertebral disc (white arrow with dotted line) and T2W hyperintense subligamentous abscess spread (solid white arrow), extending from levels T6 to T10. (b) Sagittal T1-weighted post-contrast magnetic resonance imaging of the cervical and thoracic spine revealed enhancement of an anterior extra-dural collection (white arrow with dotted line). Tissue Gene Xpert confirmed tuberculosis.

Subsequent spread of infection is beneath the anterior or posterior longitudinal ligament or through the vertebral end plates (Figure 4).^6,8^ Paraspinal and extradural soft tissue and gibbus formation are the most common findings reported. On MRI, the paraspinal or subligamentous abscesses demonstrate T2-weighted (T2W) hyperintense and T1W hypointense signal.^6,8^

**FIGURE 5:**
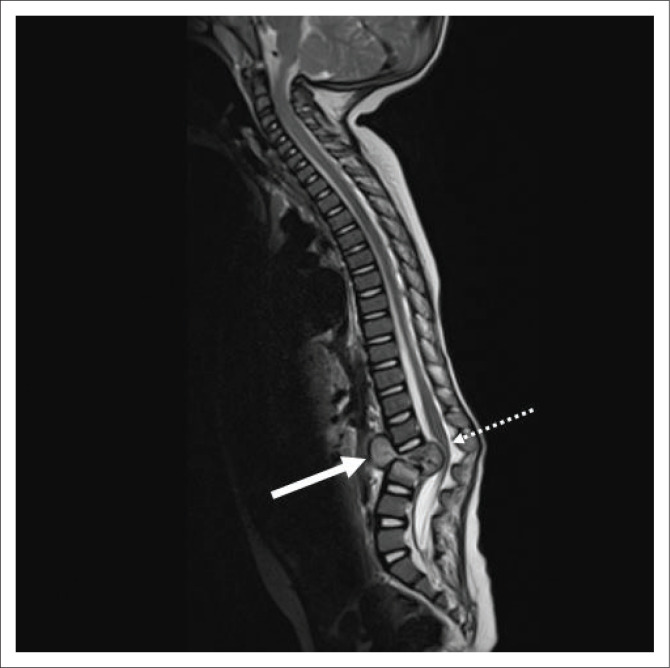
A 3-year-old girl, human immunodeficiency virus negative, presented with weight loss, acute onset of back pain in the lumbar region and refusal to walk. Chest radiograph findings were suggestive of pulmonary tuberculosis. Sagittal T2-weighted magnetic resonance imaging of the spine demonstrates near-complete destruction of the L2 vertebral body with gibbus formation at this site and T2W hyperintense subligamentous abscess (solid white arrow) extending from levels L1 to L3, with compounded compression of the conus medullaris and cauda equina nerve roots (white arrow with dotted line). Tuberculosis was confirmed on tissue GeneXpert.

Extradural abscess may cause cord compression with neurological fallout (Figure 5).^6,8^ Psoas abscess formation with possible associated calcifications is often a clue to the diagnosis.^6,8^

*Mycobacterium tuberculosis* preferentially affects end plates, with relative sparing of the intervertebral disc, whilst pyogenic infection affects the intervertebral disc during the early stage of the disease. Metastases typically involve the posterior elements more commonly with expansion of the vertebral body.

#### Intra-medullary tuberculoma

**FIGURE 6:**
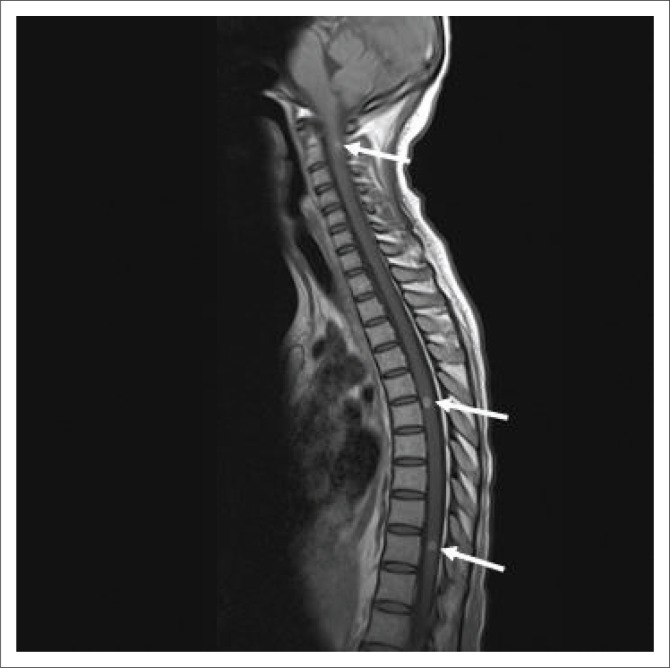
A 27-year-old human immunodeficiency virus-positive female, on antiretroviral treatment, presented with paraplegia. Sagittal T1-weighted post-contrasted magnetic resonance imaging revealed multiple round, intra-medullary rim-enhancing T1W hypointense lesions scattered throughout the spinal cord, consistent with tuberculomas (solid white arrows). Cerebrospinal fluid analysis confirmed tuberculosis.

Intra-medullary tuberculomas demonstrate signal intensities identical to intracranial tuberculomas (Figure 6).^6,7,8^

#### Arachnoiditis

Arachnoiditis is characterised by clumped, thickened, and enhancing nerve roots adherent to the dura, with CSF loculation and attenuation of the CSF spaces as typical features on both CT and MRI.^6,7^

## Head and neck tuberculosis

**FIGURE 7:**
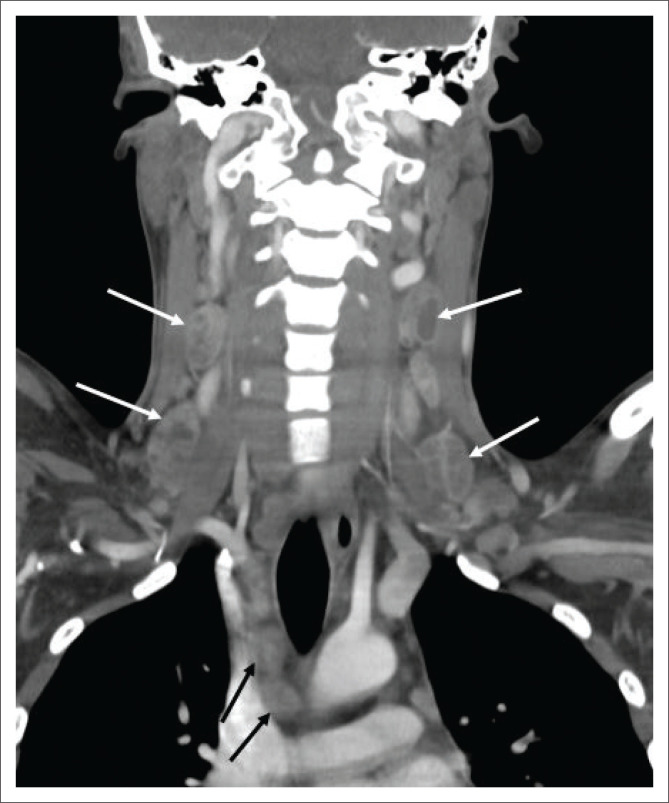
A 24-year-old human immunodeficiency virus-negative male with aplastic anaemia presented with bilateral neck swelling. Lymph node biopsy confirmed tuberculous lymphadenitis. Coronal post-contrasted computed tomography of the neck demonstrated multiple, bilateral cervical (solid white arrows) and paratracheal (solid black arrows) lymph nodes with necrotic centres consistent with tuberculous lymphadenitis.

The lymphatic system is the second most common extra-pulmonary site affected by TB in this study setting. The most common presentation is matted, painless lymphadenitis (scrofula), with only mild inflammatory superficial skin changes. Cervical lymph nodes are typically involved.^5,6,8^ Central necrosis is a typical finding and may be seen as hypoechoic lymph node centres on ultrasound and low-density central attenuation on CT, depending on the degree of caseation, with possible rim enhancement on post-contrast imaging (Figure 7).^6,8^

Paradoxical transient nodal enlargement during treatment is usually observed in HIV patients.^9^ Lymphoma and other infective causes of lymph node enlargement must be excluded, usually by fine needle aspiration or core biopsy of the involved lymph nodes.

The sinonasal cavity, larynx and glottis may also be involved, with non-specific imaging findings.^10^

## Breast tuberculosis

Breast involvement occurs rarely, with the most frequent presentation being a hard painless mass or mastalgia in a young, multiparous woman. Sinus tracts and abscesses are the associated findings.^8^

Three types of breast involvement are recognised: nodular, diffuse and sclerosing. The nodular form presents as a dense round area, which represents a caseating lesion, whilst the diffuse form leads to sinus tracts and ulceration. Fibrosis with nipple retraction is the dominant feature of the sclerosing form.^11^

## Cardiovascular tuberculosis

### Pericardium

**FIGURE 8:**
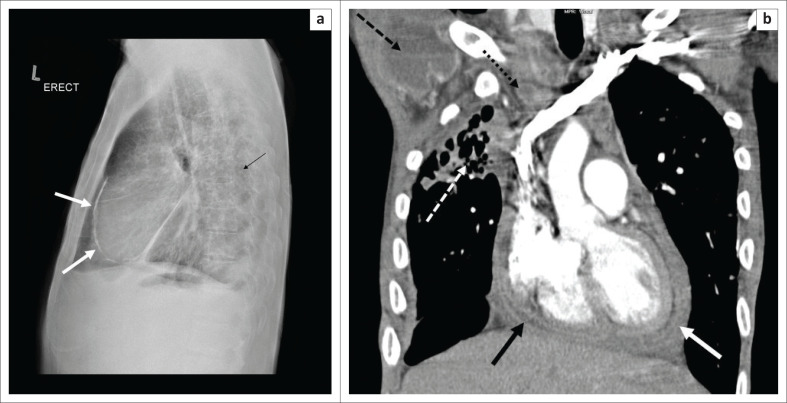
(a) A 68-year-old human immunodeficiency virus-negative male presented with features of pericarditis. GeneXpert and bronchial washings confirmed tuberculosis. A lateral chest radiograph demonstrates pericardial calcification (solid white arrows) and multiple pulmonary nodules (black arrow). (b) A 37-year-old male, human immunodeficiency virus positive with pulmonary and pericardial tuberculosis, confirmed with GeneXpert on both sputum and pericardial fluid. Coronal post-contrasted computed tomography of the chest demonstrates pericardial thickening (solid white arrow), small pericardial effusion (black arrow), large necrotic right axillary (black arrow with dashed line), smaller necrotic mediastinal (black arrow with dotted line) lymph nodes and right apical post-infective bronchiectasis (white arrow with dashed line).

Spread of infection to the pericardium may occur via haematogenous dissemination or direct thoracic lymph node extention. The typical findings include a globular cardiac configuration on chest radiography related to pericardial effusion, with late-stage pericardial calcifications (Figure 8a).

Echocardiography may demonstrate a fibrinous effusion. Slow accumulation of pericardial effusion is typically seen without tamponade. Mild pericardial thickening and enhancement may be evident on CT (Figure 8b).^8^

Constrictive pericarditis is a consequence of pericardial involvement, with typical pericardial calcifications seen at chest radiography.

### Myocardium

Although very rare, three types of involvement are recognised, which include the miliary, infiltrating interstitial and caseating nodular types.^12^

### Aortic

Secondary involvement from contiguous mediastinal lymphadenopathy, empyema, pericarditis or haematogenous dissemination. Pseudo-aneurysms may occur because of contiguous vertebral involvement.^13^

## Abdominal tuberculosis

Spread of infection may occur via ingestion of mycobacteria, haematogenous dissemination, contiguous spread from adjacent organs or lymphatic involvement.^5,6^

### Peritoneal involvement

Three types of peritoneal involvement can occur, namely, the wet, fibrotic and dry. The wet type is the most common, presenting as free ascites or loculated pockets of high-protein content fluid. The dry type leads to fibrous adhesions with mesenteric thickening. The fibrous type may manifest as omental or mesenteric masses.^5,6^

### Omentum

Omental involvement may result in a combination of omental caking or mass formation.^5^ Differential diagnosis includes carcinomatosis in the case of a known primary neoplasm or mesothelioma, if possible asbestos exposure is present.

### Lymph nodes, liver and spleen

**FIGURE 9:**
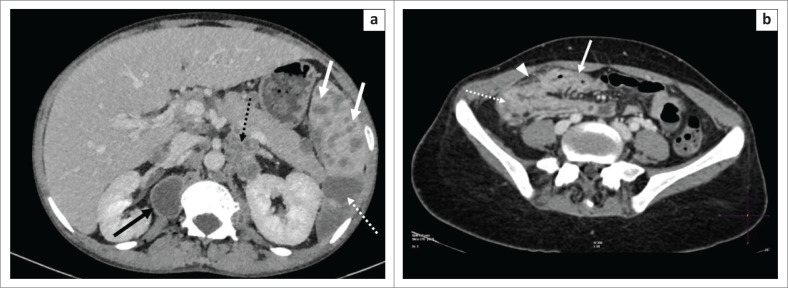
(a) A 34-year-old human immunodeficiency virus-positive female, on retroviral treatment, with previously treated multidrug-resistant tuberculosis presented with acute on chronic gastroenteritis and loss of weight. Axial post-contrasted computed tomography through the upper abdomen revealed multiple splenic micro- (solid white arrows) and macro- (white arrow with dashed line) abscesses with enlarged necrotic right paraspinal (solid black arrow) and clustered necrotic left para-aortic lymph nodes. (b) Axial slices through the pelvis of the same patient revealed diffuse, irregular thickening and enhancement of the terminal ileum (solid white arrow) and caecum (white arrow with dotted line) with peri-caecal fat stranding (white solid arrow head).

Intra-abdominal lymph nodes demonstrate the typical low-density, central caseous necrosis and rim enhancement at CT, similar to lymph node involvement in the neck or chest. Hepato-splenic involvement may be in the form of organomegaly, with micro- or rarely macroabscesses, which can calcify (Figure 9a).^5,6,8^

### Gastrointestinal tract

There is a predilection for the terminal ileum and caecum, with acute findings including mural thickening, narrowed terminal ileum and adjacent lymphadenopathy (often necrotic).^5,6,8^

A widely gaping, iliocaecal valve (Fleischner sign, Figure 9b) and a shrunken conical caecum (Figure 9b) are seen in the chronic stages. Differential diagnoses include Crohn’s disease and lymphoma.

### Adrenal glands

The adrenal glands typically demonstrate bilateral involvement. There is gland enlargement and rim enhancement with central low-density necrosis in keeping with adrenalitis.^5^ The acute gland enlargement may either resolve or lead to small, dystrophic calcified glands.

**FIGURE 10:**
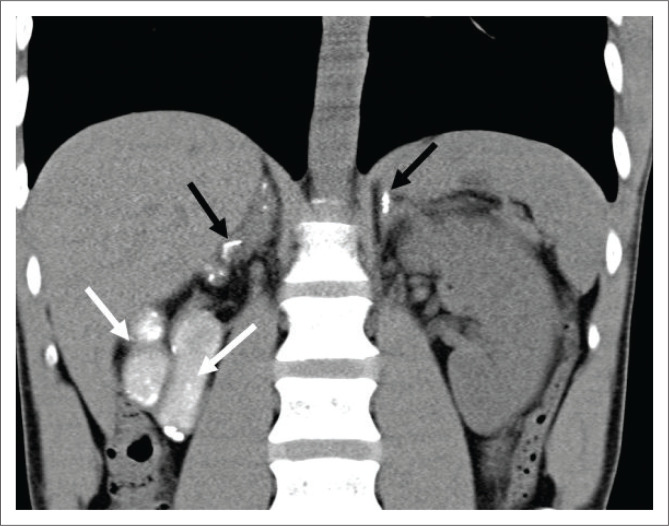
A 33-year-old male, with human immunodeficiency virus and previous tuberculosis status unknown, presented with newly diagnosed Addison’s disease. Coronal uncontrasted computed tomography of the abdomen centred over the kidneys revealed a small, echogenic, near complete calcified right kidney (‘putty kidney’; solid white arrows) and scattered calcifications throughout both adrenal glands (solid black arrows). The diagnosis of tuberculosis was made on the basis of the computed tomography findings.

Tuberculosis is the most common cause of Addison’s disease (Figure 10), and the patient may present with an acute Addisonian crisis.^5,6^

### Genito-urinary

**FIGURE 11:**
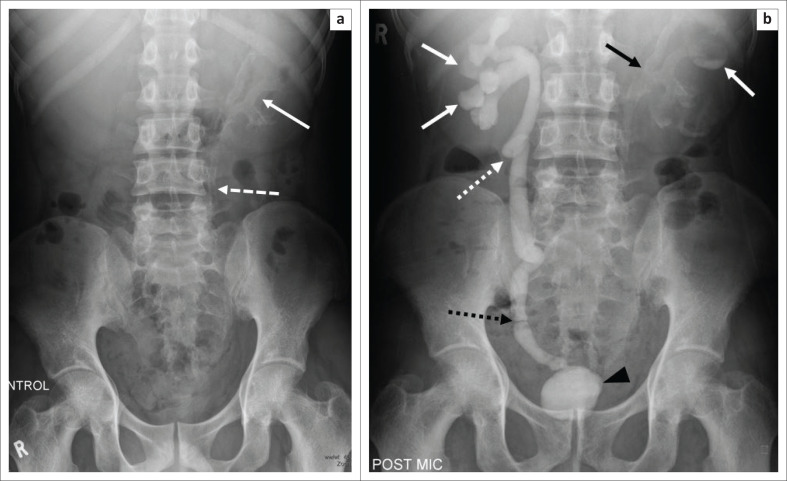
(a) A 43-year-old man known with human immunodeficiency virus and pulmonary tuberculosis, on both antiretroviral and tuberculosis treatment presented with a 3-month history of dysuria and right epididymal swelling. At intravenous pyelography, the control radiograph demonstrated diffuse urothelial calcification of the left pelvi-calyceal system (solid white arrow) and proximal left ureter (white arrow with dashed line). (b) A post-micturition view during intravenous pyelography in the same patient demonstrated bilateral blunting of the calyces (solid white arrows), a Kerr’s kink (white arrow with dotted line), focal stenosis in the distal right ureter (black arrow with dotted line), a small calibre bladder which contains residual contrast on the post-micturition view (black arrow head) and calcification of the left pelvi-calyceal system (solid black arrow).

Irregular renal cortical calcifications (Figure 10) and focal caliectasis are the common findings. Other possible findings include renal papillary necrosis, ureteric stenosis (pipe stem), ureteric calcifications (Figure 11a) and pelviureteric junction narrowing (Kerr’s kink, Figure 11b). Renal atrophy with ground glass calcifications (Putty kidney, Figure 10) are late sequelae.^5,6,8^

Bladder wall irregularity, with peripheral calcifications and a small-volume (‘thimble’) bladder, may be found, which may result in vesico-ureteric reflux with hydronephrosis related to fibrosis at the ureteric orifice.^8^

Epididymal and testicular involvement may cause epididymo-orchitis. Seminal vesicles and vas deferens may be affected with wall thickening or calcifications. Prostatic involvement may take the form of abscess or prostatitis. Diffuse dystrophic calcification is seen in the chronic form.^14^

Salpingitis, with a fallopian stricture, typically occurs at the junction of the isthmus and ampulla.^6,7^ Endometrial adhesions or synechiae formation may develop.^6,8^

## Musculoskeletal

**FIGURE 12:**
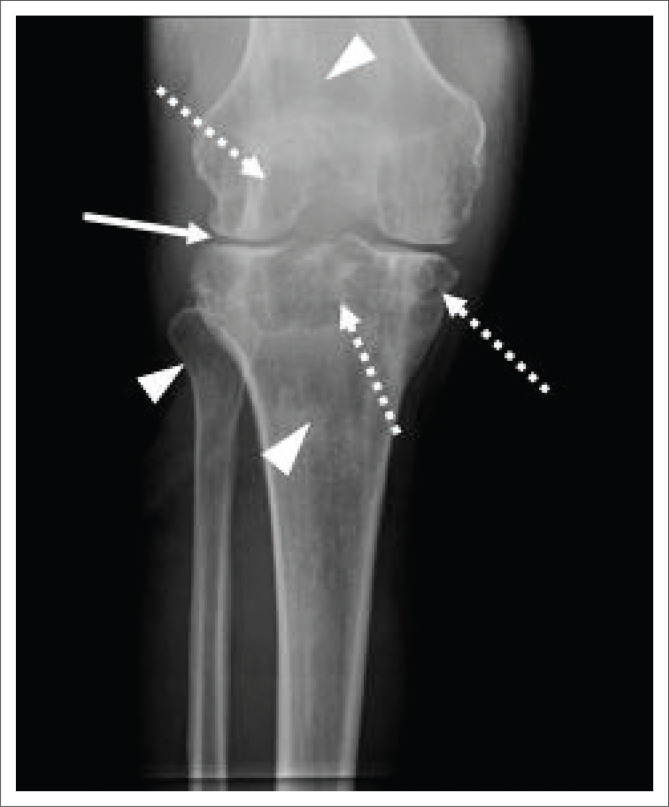
A 41-year-old male, with human immunodeficiency virus status unknown, presented with a 2-year history of chronic right knee pain and a draining sinus. Erythrocyte sedimentation rate at the initial visit was raised. Conventional radiograph of the knee demonstrates joint space narrowing (solid white arrow), subchondral cyst formation (white arrows with dotted lines) and juxta-articular osteopenia (white arrow heads). The constellation of findings is known as the Phemister triad.

**FIGURE 13:**
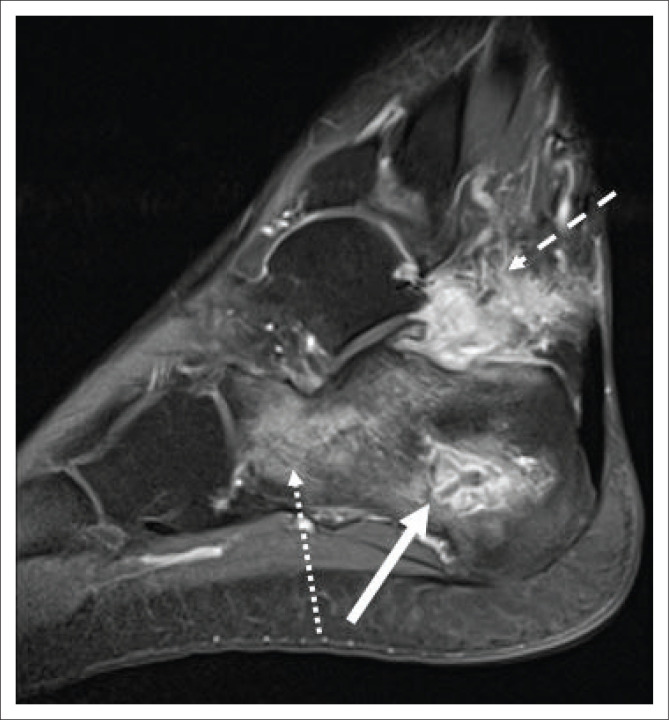
A 23-year-old male, human immunodeficiency virus status unknown, presented with left heel pain and a draining sinus. Sagittal T1-weighted fat sat, post-contrasted magnetic resonance imaging of the left hind foot demonstrates a small rim-enhancing collection in the calcaneus (solid white arrow) with enhancing inflammatory changes in the remainder of the calcaneus (white arrow with dotted line) and surrounding soft tissues (white arrow with dashed line). A draining sinus was demonstrated on T2W sequences. Tuberculosis was confirmed on Gene Xpert of calcaneal tissue.

**FIGURE 14:**
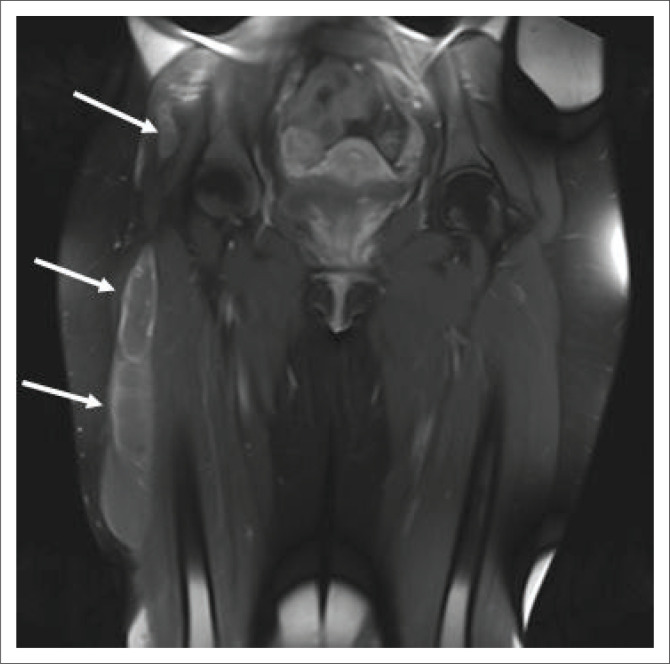
A 20-year-old human immunodeficiency virus-negative female presented with chronic hip pain, which progressively worsened over 3 months. Coronal T1-weighted fat sat post-contrasted magnetic resonance imaging of the pelvis demonstrates rim-enhancing masses within the greater trochanter and intertrochanteric space (images not included), which communicates with the right trochanteric bursa. Rim-enhancing abscesses (solid white arrows) extend from the bursa, inferiorly underneath the ilio-tibial band. Tuberculous osteitis with overlying bursitis and cold abscess formation was confirmed on tissue Gene expert.

**FIGURE 15:**
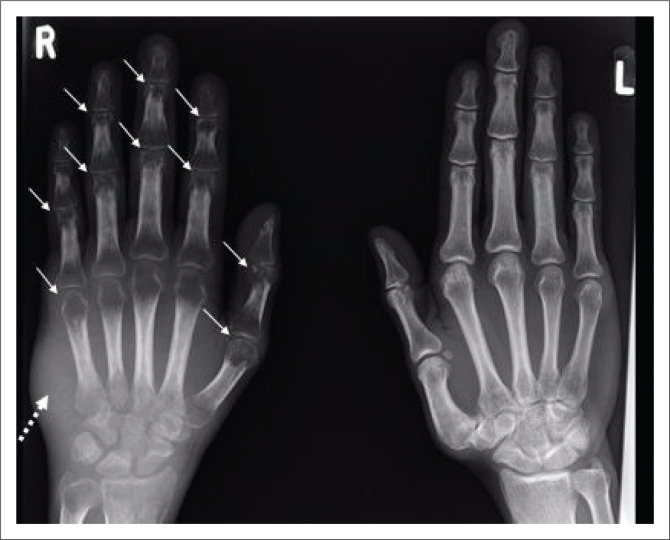
A 40-year-old human immunodeficiency virus-negative male presented with a 2-month history of unilateral right hand swelling. A conventional radiograph of the hands demonstrates diffuse periarticular osteopenia (solid white arrows) and right-sided soft tissue swelling (white arrow with dotted line). No erosions or joint space narrowing were observed. Synovial biopsy confirmed tuberculous tenosynovitis.

Monoarthritis of weight-bearing joints is common.^6,8^ Patterns of involvement include spondylitis, tuberculous arthritis (Figure 12 – knee), osteitis or osteomyelitis (Figure 13), soft tissue involvement, bursitis (Figure 14), tenosynovitis (Figure 15) and dactylitis.^5,6,8^ Periarticular osteopenia, marginal erosions and subtle joint space loss are seen with articular involvement and are called the Phemister triad (Figure 12).^5,6,8^ A recent case series by Swarap et al.,^15^ demonstrates atypical sites of musculoskeletal involvement, and emphasises the need for maintaining a high index of suspicion in order to make the correct diagnosis.

## Conclusion

Tuberculosis remains a common diagnosis made on a daily basis by radiologists in South Africa, and hence, is known to be a silent pandemic when misdiagnosed. Extra-pulmonary TB remains underdiagnosed, with patients often presenting at advanced stages of the disease with extensive destruction of the organ system(s) involved, which is mainly because of the stigma and its association with HIV infection.

Tuberculosis is known as the great mimicker and remains the leading cause of communicable-disease-related deaths in our country. It must be considered as a differential when evaluating patients with sequelae of chronic infection in all pandemic areas.
